# Geography of suicide in Japan: spatial patterning and rural–urban differences

**DOI:** 10.1007/s00127-020-01978-7

**Published:** 2020-11-07

**Authors:** Eiji Yoshioka, Sharon J. B. Hanley, Yukihiro Sato, Yasuaki Saijo

**Affiliations:** 1 Department of Social Medicine, Asahikawa Medical University, Midorigaoka-higashi 2-1-1-1, Asahikawa, Hokkaido 078-8510 Japan; 2 Department of Obstetrics and Gynecology, Hokkaido University Graduate School of Medicine, Kita 15, Nishi 7, Kita-ku, Sapporo, 060-8638 Japan

**Keywords:** Suicide, Geographical variation, Level of rurality/urbanity, Spatial analysis, Bayesian hierarchical models

## Abstract

**Purpose:**

There are notable geographic variations in incidence rates of suicide both in Japan and globally. Previous studies have found that rurality/urbanity shapes intra-regional differences in suicide mortality, and suicide risk associated with rurality can vary significantly by gender and age. This study aimed to examine spatial patterning of and rural–urban differences in suicide mortality by gender and age group across 1887 municipalities in Japan between 2009 and 2017.

**Methods:**

Suicide data were obtained from suicide statistics of the Ministry of Health, Labour and Welfare in Japan. We estimated smoothed standardized mortality ratios for suicide for each of the municipalities and investigated associations with level of rurality/urbanity using Bayesian hierarchical models before and after adjusting for socioeconomic characteristics.

**Results:**

The results of the multivariate analyses showed that, for males aged 0–39 and 40–59 years, rural residents tended to have a higher suicide risk compared to urban ones. For males aged 60+ years, a distinct rural–urban gradient in suicide risk was not observed. For females aged 0–39 years, a significant association between suicide risk and rurality was not observed, while for females aged 40–59 years and females aged 60 years or above, the association was a U-shaped curve.

**Conclusion:**

Our results showed that geographical distribution of and rural–urban differences in suicide mortality in Japan differed substantially by gender and age. These findings suggest that it is important to take demographic factors into consideration when municipalities allocate resources for suicide prevention.

## Introduction

Suicide is a major public health issue in Japan. According to mortality data from vital statistics in Japan [1], the crude suicide rate in 2017 was 16.4 per 100,000 population (23.6 for males and 10.1 for females), making suicide the 9th leading cause of death in Japan. There are notable geographic variations in the incidence of suicide worldwide. According to one WHO report [2], national suicide rates range from 0.4 to 44.2 per 100,000 population. Within the same country, incidence of suicide also varies between regions and distinct features exists in geographic distribution [3–5].

Detailed spatial analyses are considered to be useful for investigating the geographic pattern of suicide [3, 6]. Small geographic units have more internal homogeneity than large units and their aggregate socioeconomic characteristics are more likely to reflect the nature of social environment, where people live [4, 6]. However, in areas with small populations the small number of deaths can result in unreliable estimates. A Bayesian hierarchical Poisson regression model can be used to address this problem of uncertainty in estimates in small-area analyses [7, 8], and has already been used successfully in previous studies on suicide [3–5, 9–11]. Findings from a study analyzing gender-/age-specific spatial patterning of suicide using data for small geographic units would make it possible to identify regions which warrant particular attention in terms of interventions for suicide prevention, and are thus important for policy makers and public health officers.

Previous studies have found that rurality/urbanity shapes intra-regional differences in suicide rates [4, 10, 12–18]. Some aspects of rural circumstances, such as geographical and interpersonal isolation and lack of access to care have been suggested to be associated with an increased risk of suicide [19]. While a recent review article suggested that rural rates of suicidal behavior and death by suicide were often higher than those in urban areas [19], some studies have reported that the association between suicide risk and rurality/urbanicity varies significantly by gender and age [15, 18]. Concerning gender differences, previous studies from Australia, Scotland, and USA reported that male rural residents were at higher risk but females were not [10, 16, 20]. On the other hand, in the Netherland, Portugal, South Korea, and Taiwan both male and female rural residents were shown to be at higher risk [4, 5, 13, 21]. Furthermore, findings have not been consistent on rural–urban differences in area suicide risk by age in previous studies. Ecological studies in the Netherland, South Korea and Taiwan showed that, regardless of age, suicide risk tended to be higher in rural areas than in urban areas [4, 13, 21]. On the other hand, researches carried out in Austria, Denmark, New Zealand, and the UK found that rural–urban differences in suicide risk varied by age [14, 15, 18, 22]. For example, ecological research in Austria showed that suicide rates tended to be significantly higher in rural areas than urban areas among people aged 10–24 and 40–64 years, but no such associations were found for the other age groups [14]. In Japan, previous studies have reported that for men, rural residents had a higher suicide risk compared with urban residents, but not for women [23–25]. However, almost all of these studies used mortality data for rather large geographic units (e.g., prefecture), and age-specific analyzes have not been fully explored.

In this study, we used Bayesian hierarchical models to provide a detailed picture of spatial pattern from the period 2009–2017. We used pooled 9-year data, instead of 1-year data, to reduce the high level of variability and instability involved in estimations of suicide rates for small areas, potentially providing a more accurate picture of the geographical distribution of suicide. We then examined differences in suicide risk in relation to levels of rurality/urbanicity, defined according to population density in the area.

## Methods

### Suicide and population data

Suicide data between 2009 and 2017 were obtained from the suicide statistics of the Ministry of Health, Labour and Welfare in Japan [26], and included information on the number of suicides by gender, age, and municipality location. There are two statistics on suicide in Japan. One is the cause-of-death statistics included in the vital statistics, and the other is the suicide statistics used in this study. ICD-10 codes are used in the cause-of-death statistics but not in the suicide statistics. We used the suicide statistics instead of the cause-of-death statistics in this study, because we could not obtain the cause-of-death statistics including the number of suicides by age for each municipality. In Japan, the cause of death and manner of death are confirmed by a medical doctor. And if the manner of death is determined to be unnatural death (accident, suicide, homicide, or undetermined), it must be reported to the police. The suicide statistics used in this study are based on data on unnatural death collected by the police agency. Each suicide is assigned to a municipality (median population = 30,534) based on residential address before death. In this study, the units of analyses were municipalities. The category of municipality in Japan consists of “special wards of the Tokyo Metropolis,” “cities,” “towns,” and “villages.” In addition, 20 large cities (cities designated by ordinance) consist of several wards. These wards were also used as municipalities in this study. Because three of the cities designated by ordinance (Kumamoto, Okayama, Sagamihara) were subdivided into wards after January 2009, these cities were aggregated in this study. Therefore, although there were 1896 municipalities in Japan in 2017, suicide data were grouped into 1887 aggregated municipalities. Population data for each of the municipalities in Japan in each year were obtained from demographic surveys based on the nation’s domiciliary registration system [27].

### Rurality/urbanity

We categorized the 1887 municipalities of Japan into ten deciles of rurality/urbanity based on population density in 2010. Descriptive statistics of the ten categories of rurality/urbanity are presented in Table 1. Population density was obtained from the 2010 Census [28]. Population density reflects the relatively fixed characteristics of each municipality in Japan during the research period. Furthermore, the Pearson correlation coefficient for municipal population density between 2010 and 2015 was 0.999.

**Table 1:** Descriptive statistics of 10 categories of rurality/urbanity base on population density in Japan in 2010

	Number of municipalities	Range of population density (person per km^2^)	Percentage of the overall population in Japan in 2010 (%)
Most urban	189	4852.3–21,898.3	29.3
2nd most urban	189	1654.6–4840.4	18.2
3rd most urban	189	796.9–1636.0	16.7
4th most urban	188	409.8–795.8	10.6
5th most urban	189	248.2–409.1	8.9
5th most rural	189	151.1–248.1	6.5
4th most rural	188	92.1–150.8	4.3
3rd most rural	189	52.0–91.1	3.2
2nd most rural	189	21.9–51.9	1.6
Most rural	188	1.6–21.8	0.7

### Covariates

Data on the following four socioeconomic characteristics at the municipality level were extracted from the 2010 census and considered as possible confounders [28]: single-person households (% of single-person households) [22, 29]; unmarried adults (% of unmarried adults) [22, 29]; unemployment rate (% of people in paid employment aged 15+ , excluding those in school or higher education, housewives, retirees, and those unable to work for health reasons) [6, 30]; educational attainment (% of people aged 35–64 years with college or higher education) [6, 30]. Among the four characteristics above, single-person households and educational attainment were log-transformed, because distributions of the raw values were skewed. Finally, all four characteristics were standardized (z scores).

### Statistical analysis

All analyses were stratified by gender. For each municipality, we calculated ‘raw’ (unsmoothed) standardized mortality ratios (SMRs: the ratio of the observed to the expected number of suicides) for inhabitants during the period 2009–2017. Expected suicides were calculated by multiplying the national gender-and age-specific suicide rates (in 10-year age-bands) by the corresponding gender-and age-specific population in each municipality. SMRs for males and females under the age of 40 years, 40–59 years and 60 years or above were also calculated separately. Geographic variations in suicide rates were presented using differences over the middle 90% of SMRs (i.e., the ratios between values at 95% and 5%), as the extreme values at both ends of the distribution are likely to be unreliable estimates.

Bayesian hierarchical models were used to estimate the ‘smoothed’ SMR for each municipality and investigate associations among level of rurality/urbanity and suicide rates. These were based on Poisson regression models with random effects allowing for both non-structural variability (heterogeneity across all areas in the study region) and structural variability (autocorrelation between neighboring areas) [7, 31, 32]. In the models used, an intrinsic conditional autoregressive prior distribution was assigned to the random effect for structural variability, while the random effect for non-structural variability was represented using independent normal distributions. The default prior distributions were specified for the model parameters [33]. Sensitivity tests with altered hyperparameters did not change the results, confirming the robustness of the results. Sets of municipalities that share a border were defined as neighboring areas. Concerning island areas, sets of municipalities that have a regular sea route were defined as neighboring areas, therefore all municipalities had some neighboring areas. Associations with the level of rurality/urbanity were also examined before and after controlling for socioeconomic characteristics in the models, ‘Residual’ SMRs after controlling for the effects of rurality and the socioeconomic characteristics were estimated and mapped to investigate the spatial patterning of residual variation which could not be accounted for by the studied variables. The models were estimated with integrated nested Laplace approximation [34, 35]. Statistical analyses of the models were carried out using the R-INLA library (18.07.12) in R-3.5.3. All other statistical analyses were performed using Stata statistical software, version 15.1, for Macintosh (StataCorp, College Station, TX, USA).

SMRs were mapped using seven categories that are symmetrical on the logarithmic scale (< 0.50, 0.50–< 0.67, 0.67–< 0.90, 0.90–< 1.10, 1.10–< 1.50, 1.50–< 2.00, and ≥ 2.00). Brown, blue and pale yellow with varying degrees of lightness were used to present those higher (brown) and lower (blue) than the middle category (pale yellow), respectively. All maps were produced using QGIS Version 2.18.15 for Macintosh.

## Results

There were 240,673 suicides (males: 166,859 [69.3%]) in Japan between 2009 and 2017. Of these, 2699 (1.1%) suicides were excluded from the analysis, because address or age data were unavailable. Of the 237,974 suicides included in the analysis, 164,432 (69.1%) were male.

**Fig. 1:**
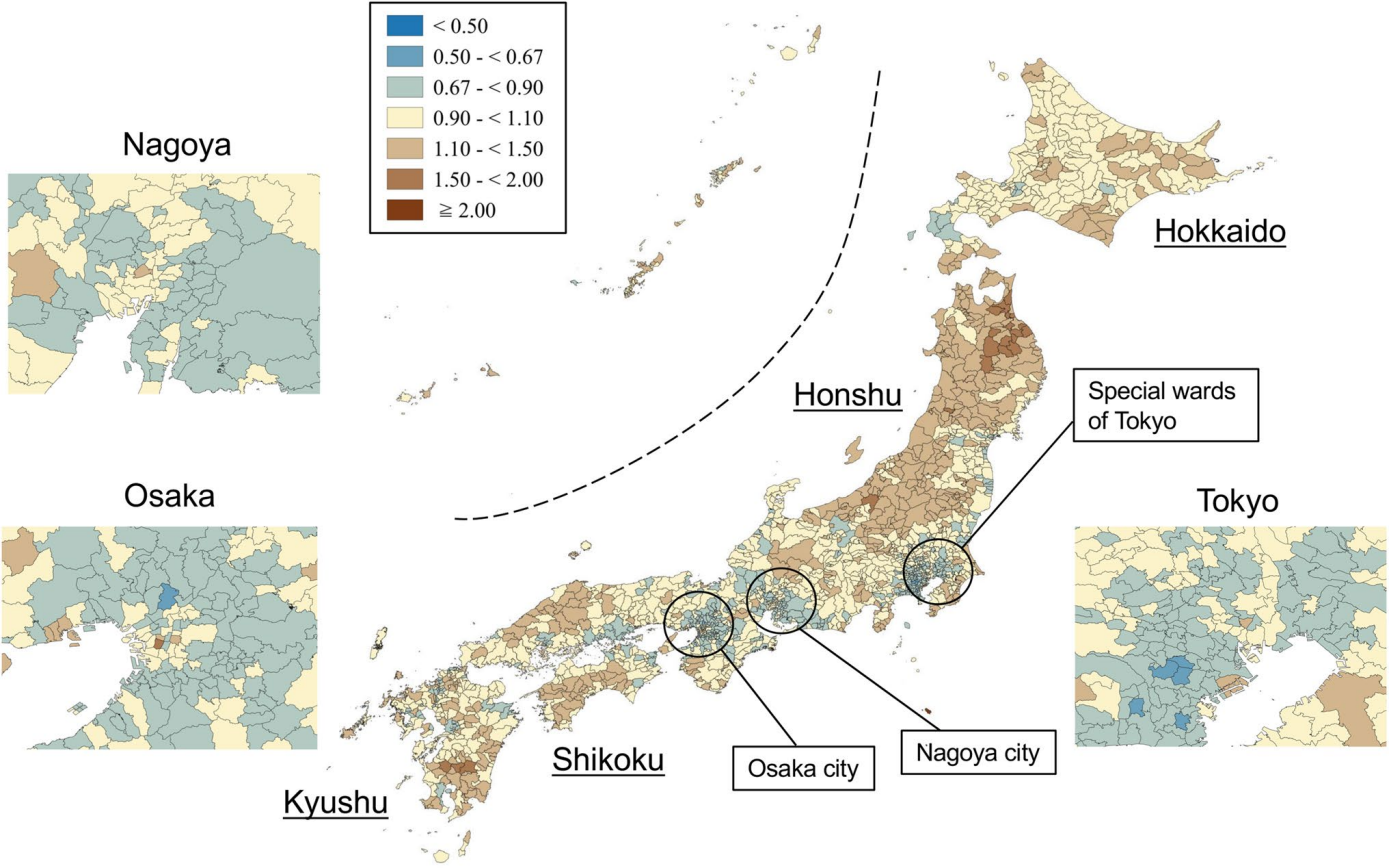
Maps of smoothed standardized mortality ratios (sSMRs) for suicide in males of all ages across 1887 municipalities in Japan, 2009–2017

**Fig. 2:**
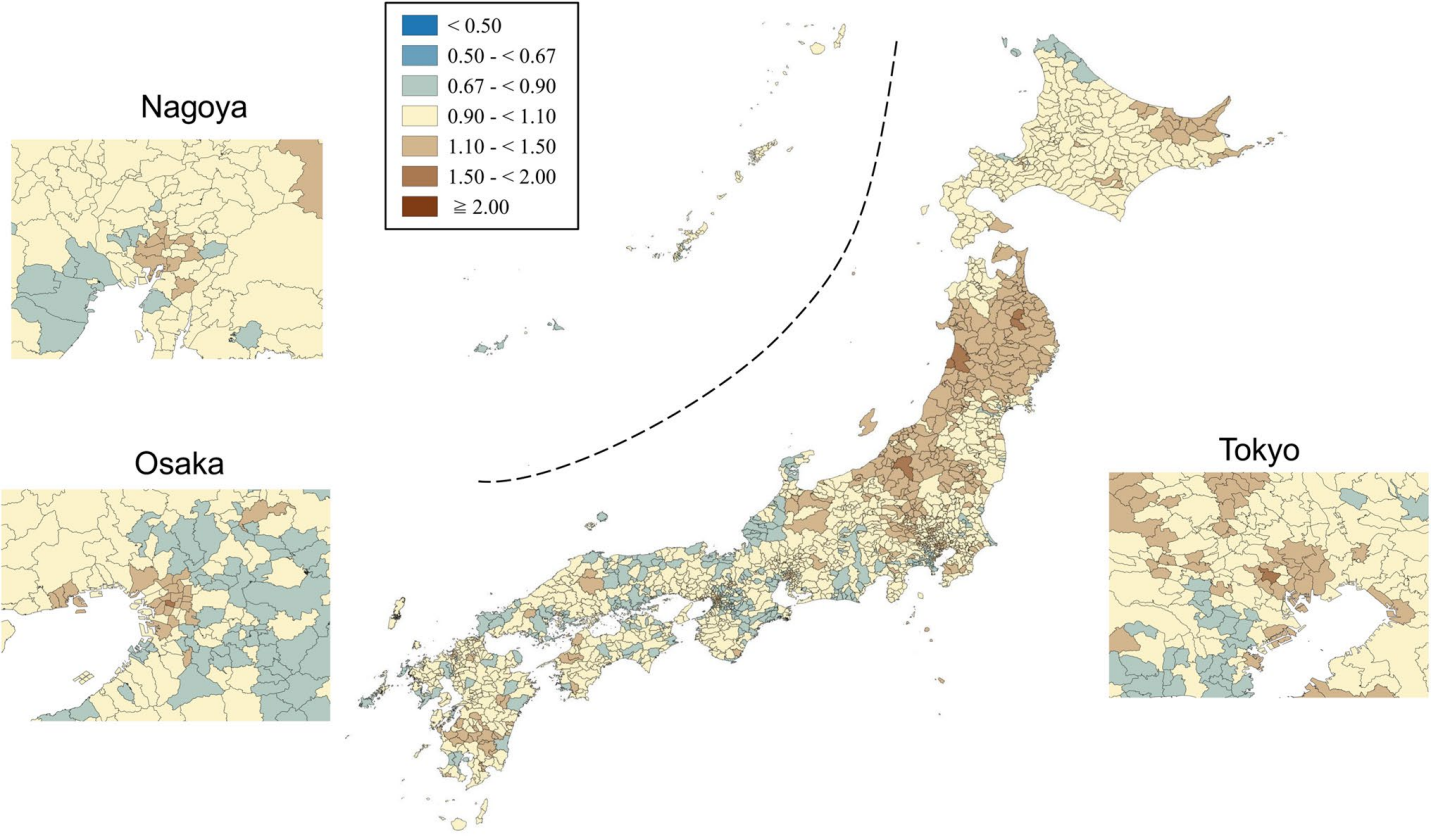
Maps of smoothed standardised mortality ratios (sSMRs) for suicide in females of all ages across 1887 municipalities in Japan, 2009–2017

Figures 1 and 2 show the geographic distribution of smoothed SMRs for suicide in males and females of all ages, respectively. Japan consists of four main islands: Hokkaido, Honshu, Shikoku, and Kyushu, going from east to west. Three metropolitan areas (Tokyo, Osaka, and Nagoya) in Japan are shown in the enlarged maps. Smoothed SMRs ranged from 0.62 to 2.55 (90% range 0.79–1.31, a 1.7-fold difference) for males of all ages and from 0.70 to 1.74 (90% range 0.85–1.25, a 1.5-fold difference) for females of all ages. The municipalities with a high risk of suicide for males of all ages were clustered in the northern part of Honshu Island (Tohoku region), the southern part of Kyushu Island, the southern part of Shikoku Island, some coastal areas facing the Sea of Japan, and some areas of Hokkaido Island. The three metropolitan areas appeared to have a lower suicide risk for males of all ages. The municipalities with a high risk of suicide for females of all ages were clustered in the Tohoku region, the southern part of Kyushu Island, and the eastern part of Hokkaido Island. In contrast to males, the municipalities with a high risk of suicide for females of all ages appeared to cluster in the metropolitan areas.

Table 2 shows age-specific estimates of rate ratios of suicide for males by level of rurality before and after adjusting for socioeconomic characteristics. For males of all ages, there was a roughly increasing trend in rate ratios of suicide across levels of rurality/urbanity both before and after the adjustment, suggesting that rural municipalities had larger rate ratios than urban ones. Subgroup analyses by age showed that, for males aged 0–39 and 40–59 years, there was a roughly increasing trend in rate ratios across levels of rurality/urbanity before and after the adjustment. For males aged 60+ years, a roughly increasing trend was observed in the unadjusted model, but the rate ratios were substantially reduced across levels of rurality/urbanity after the adjustment, resulting in significant associations only in the 2nd and 3rd rural municipalities.

**Table 2:** Rate ratios (and 95% credible intervals) of suicide for males by rural–urban continuum and age before and after adjusting for socioeconomic characteristics, Japan, 2009–2017

	All ages				0–39 years				40–59 years				60+ years			
	Unadjusted		Adjusted^a^		Unadjusted		Adjusted^a^		Unadjusted		Adjusted^a^		Unadjusted		Adjusted^a^	
	RR	(95%CI)	RR	(95%CI)	RR	(95%CI)	RR	(95%CI)	RR	(95%CI)	RR	(95%CI)	RR	(95%CI)	RR	(95%CI)
Most urban	Reference		Reference		Reference		Reference		Reference		Reference		Reference		Reference	
2nd most urban	0.99	(0.96, 1.03)	1.03	(1.00, 1.06)	1.02	(0.97, 1.06)	1.07*	(1.02, 1.12)	1.00	(0.96, 1.05)	1.00	(0.96, 1.05)	0.97	(0.92, 1.02)	0.99	(0.95, 1.04)
3rd most urban	1.04	(1.00, 1.08)	1.05*	(1.02, 1.09)	1.06*	(1.01, 1.11)	1.13*	(1.07, 1.18)	1.07*	(1.02, 1.12)	1.07*	(1.02, 1.12)	0.99	(0.94, 1.05)	0.97 (0.92, 1.02)	
4th most urban	1.07*	(1.03, 1.12)	1.05*	(1.01, 1.09)	1.06*	(1.01, 1.12)	1.13*	(1.07, 1.20)	1.13*	(1.07, 1.19)	1.13*	(1.07, 1.19)	1.02	(0.96, 1.08)	0.95	(0.90, 1.01)
5th most urban	1.14*	(1.09, 1.20)	1.10*	(1.06, 1.15)	1.12*	(1.05, 1.18)	1.19*	(1.12, 1.27)	1.21*	(1.15, 1.28)	1.21*	(1.15, 1.28)	1.10*	(1.04, 1.17)	1.00	(0.94, 1.06)
5th most rural	1.20*	(1.14, 1.25)	1.13*	(1.08, 1.18)	1.15*	(1.08, 1.22)	1.22* (1.14, 1.30)	1.25*	(1.18, 1.32)	1.25*	(1.18, 1.32)	1.17*	(1.10, 1.25)	1.03	(0.97, 1.10)	
4th most rural	1.24*	(1.19, 1.30)	1.16*	(1.11, 1.21)	1.17*	(1.10, 1.25)	1.26*	(1.16, 1.35)	1.29*	(1.22, 1.37)	1.29*	(1.22, 1.37)	1.23*	(1.15, 1.32)	1.06	(0.99, 1.14)
3rd most rural	1.29*	(1.23, 1.35)	1.18*	(1.12, 1.24)	1.17*	(1.08, 1.26)	1.24*	(1.14, 1.34)	1.36*	(1.28, 1.45)	1.36*	(1.28, 1.45)	1.30*	(1.21, 1.39)	1.09*	(1.02, 1.17)
2nd most rural	1.34*	(1.27, 1.41)	1.21*	(1.14, 1.28)	1.25*	(1.14, 1.37)	1.33*	(1.20, 1.47)	1.41*	(1.30, 1.52)	1.41*	(1.30, 1.52)	1.32*	(1.22, 1.43)	1.09*	(1.00, 1.18)
Most rural	1.33*	(1.25, 1.42)	1.16*	(1.09, 1.25)	1.47*	(1.30, 1.66)	1.53*	(1.33, 1.75)	1.32*	(1.19, 1.46)	1.32*	(1.19, 1.46)	1.27*	(1.16, 1.40)	1.02	(0.92, 1.14)

*RR* rate ratios, *CI* credible interbals

*p value < 0.05

^a^Adjusted for single-person households, unmarried adults, unemployment rate, and educational attainment

Table 3 shows age-specific estimates of rate ratios of suicide for females by level of rurality before and after adjusting for socioeconomic characteristics. For females of all ages, the results suggested that the association between suicide risk and rurality was U-shaped with a higher risk of suicide in rural and urban municipalities and a lower risk of suicide in ones of the intermediate categories both before and after the adjustment. For females aged 0–39 years, there was a roughly decreasing trend in rate ratios of suicide across levels of rurality/urbanity in the unadjusted model, showing that rural municipalities had smaller rate ratios than urban ones. However, the significant associations at all levels of rurality disappeared after the adjustment. For females aged 40–59 years, the association between suicide risk and rurality was a U-shaped curve both before and after the adjustment. For females aged 60 + years, there was a roughly increasing trend in rate ratios across levels of rurality/urbanity in the unadjusted model. However, the rate ratios across levels of rurality reduced substantially after the adjustment, resulting in a U-shaped curve. In Appendix Table 4, the rate ratios of suicide among the total Japanese population by rurality/urbanity between 2009 and 2017 are presented. The results indicate a roughly increasing trend in rate ratios of suicide across levels of rurality/urbanity both before and after adjustment, similar to the results for males of all ages.

**Table 3:** Rate ratios (and 95% credible intervals) of suicide for females by rural–urban continuum and age before and after adjusting for socioeconomic characteristics, Japan, 2009–2017

	All ages				0–39 years				40–59 years				60 + years			
	Unadjusted		Adjusted^a^		Unadjusted		Adjusted^a^		Unadjusted		Adjusted^a^		Unadjusted		Adjusted^a^	
	RR	(95%CI)	RR	(95%CI)	RR	(95%CI)	RR	(95%CI)	RR	(95%CI)	RR	(95%CI)	RR	(95%CI)	RR	(95%CI)
Most urban	Reference		Reference		Reference		Reference		Reference		Reference		Reference		Reference	
2nd most urban	0.92*	(0.89, 0.96)	0.95*	(0.92, 0.99)	0.88*	(0.82, 0.93)	0.99	(0.93, 1.05)	0.93*	(0.88, 0.98)	0.99	(0.95, 1.04)	0.96	(0.91, 1.01)	0.94*	(0.88, 0.99)
3rd most urban	0.90*	(0.86, 0.94)	0.92*	(0.88, 0.97)	0.83*	(0.77, 0.89)	0.97	(0.90, 1.04)	0.89*	(0.84, 0.94)	0.94*	(0.89, 1.00)	0.95	(0.89, 1.02)	0.90*	(0.84, 0.96)
4th most urban	0.90*	(0.86, 0.95)	0.91*	(0.86, 0.96)	0.78*	(0.72, 0.85)	0.94	(0.86, 1.02)	0.85*	(0.80, 0.91)	0.90*	(0.84, 0.96)	1.00	(0.94, 1.08)	0.92*	(0.85, 0.99)
5th most urban	0.94*	(0.89, 0.99)	0.93*	(0.88, 0.98)	0.81*	(0.74, 0.88)	0.99	(0.90, 1.08)	0.91*	(0.84, 0.97)	0.94	(0.88, 1.01)	1.03	(0.96, 1.11)	0.91*	(0.84, 0.98)
5th most rural	0.92*	(0.87, 0.97)	0.90*	(0.85, 0.95)	0.78*	(0.70, 0.85)	0.95	(0.86, 1.06)	0.82*	(0.76, 0.89)	0.84*	(0.77, 0.91)	1.05	(0.98, 1.13)	0.92*	(0.84, 0.99)
4th most rural	0.96	(0.90, 1.01)	0.93*	(0.87, 0.99)	0.79*	(0.71, 0.88)	1.00	(0.89, 1.13)	0.88*	(0.80, 0.96)	0.90*	(0.82, 0.99)	1.07	(0.99, 1.16)	0.91*	(0.83, 0.99)
3rd most rural	1.03	(0.97, 1.10)	0.99	(0.92, 1.06)	0.78*	(0.69, 0.88)	0.98	(0.85, 1.12)	0.89*	(0.81, 0.98)	0.89*	(0.80, 0.99)	1.20*	(1.11, 1.30)	1.00	(0.91, 1.10)
2nd most rural	1.08*	(1.00, 1.16)	1.02	(0.94, 1.10)	0.77*	(0.65, 0.90)	0.97	(0.81, 1.16)	0.97	(0.85, 1.09)	0.97	(0.84, 1.10)	1.25*	(1.14, 1.36)	1.00	(0.90, 1.12)
Most rural	1.08	(0.98, 1.18)	0.99	(0.89, 1.10)	0.76*	(0.59, 0.96)	0.93	(0.71, 1.19)	0.94	(0.78, 1.12)	0.90	(0.74, 1.09)	1.24*	(1.11, 1.39)	0.97	(0.85, 1.11)

*RR* rate ratios, *CI* credible interbals

*p value < 0.05

^a^Adjusted for single-person households, unmarried adults, unemployment rate, and educational attainment

**Table 4:** Rate ratios (and 95% credible intervals) of suicide among the total Japanese population by rural–urban continuum between 2009 and 2017

	Unadjusted		Adjusted^a^	
Rural–Urban continuum	RR	(95%CI)	RR	(95%CI)
Most urban	Reference		Reference	
2nd most urban	0.97*	(0.93, 1.00)	1.00	(0.97, 1.03)
3rd most urban	0.99	(0.95, 1.03)	1.01	(0.97, 1.04)
4th most urban	1.02	(0.98, 1.06)	1.00	(0.97, 1.04)
5th most urban	1.08*	(1.03, 1.12)	1.04*	(1.00, 1.08)
5th most rural	1.10*	(1.06, 1.15)	1.05*	(1.01, 1.09)
4th most rural	1.15*	(1.10, 1.20)	1.08*	(1.03, 1.13)
3rd most rural	1.20*	(1.15, 1.25)	1.11*	(1.06, 1.16)
2nd most rural	1.25*	(1.19, 1.31)	1.14*	(1.08, 1.20)
Most rural	1.25*	(1.18, 1.32)	1.11*	(1.04, 1.18)

*RR* rate ratios, *CI* credible interbals

*p value < 0.05

^a^Adjusted for single-person households, unmarried adults, unemployment rate, and educational attainment

Appendix Figs. 3, 4, 5, 6, 7, 8 show maps of smoothed standardized SMRs for suicide in males and females aged 0–39, 40–59, and 60+ years. The maps indicate that the geographical distribution of suicide in Japan between 2009 and 2017 differed by gender and age with several regional clusters specific to some gender and age groups but not others. For example, Tohoku region had a higher suicide risk for males of all the three age groups and females aged 60+ years. The western part of Honshu island, the southern part of Shikoku island, and the southern part of Kyushu island had a higher risk for males aged 40–59 and 60+ years and females aged 60+ years. For females aged 0–39 years, high suicide risk municipalities appeared to cluster in the three metropolitans and other big cities, such as Sapporo and Sendai. For females aged 40–59 years, high suicide risk municipalities appeared to cluster in the central areas of Tokyo. 90% ranges of smoothed SMRs were 0.84–1.21 (1.4-fold difference) for males aged 0–39 years, 0.80–1.28 (1.6-fold difference) for males aged 40–59 years, 0.78–1.39 (1.8-fold difference) for males aged 60+ years, 0.87–1.24 (1.4-fold difference) for females aged 0–39 years, 0.90–1.15 (1.3-fold difference) for females aged 40–59 years, and 0.80–1.37 (1.7-fold difference) for females aged 60+ years.

**Fig. 3:**
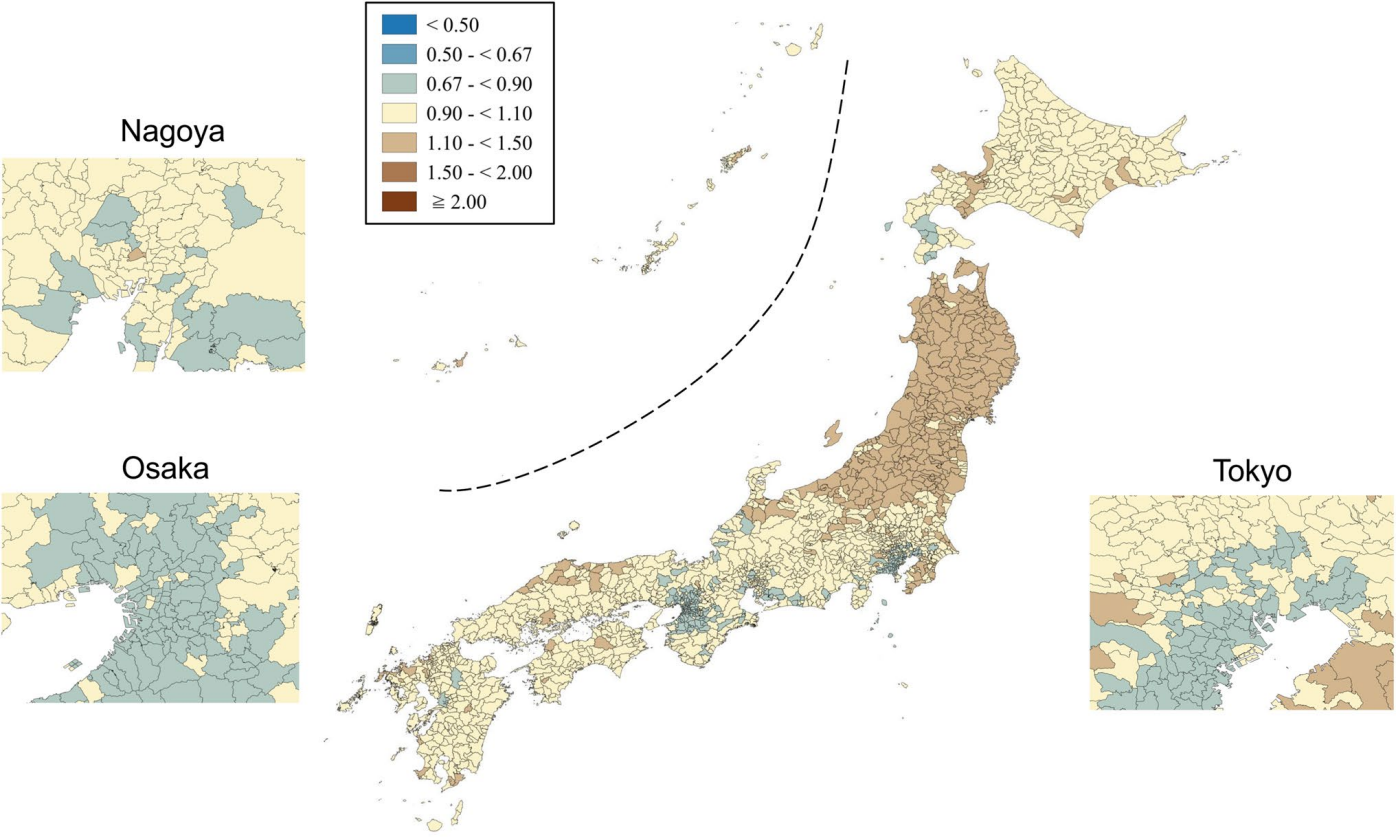
Maps of smoothed standardized mortality ratios (sSMRs) for suicide in males aged 0–39 years across 1887 municipalities in Japan, 2009–2017

**Fig. 4:**
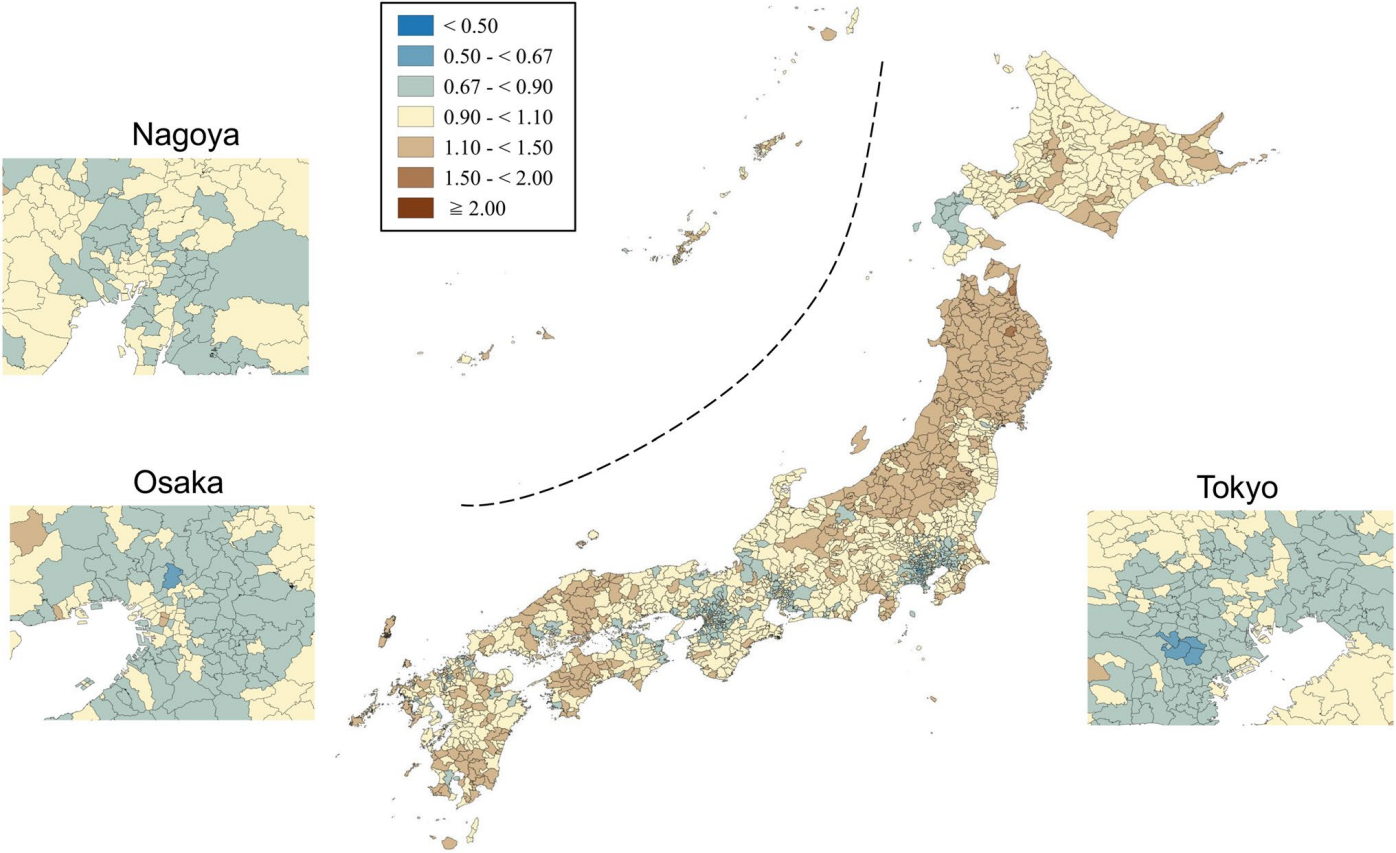
Maps of smoothed standardized mortality ratios (sSMRs) for suicide in males aged 40–59 years across 1887 municipalities in Japan, 2009–2017

**Fig. 5:**
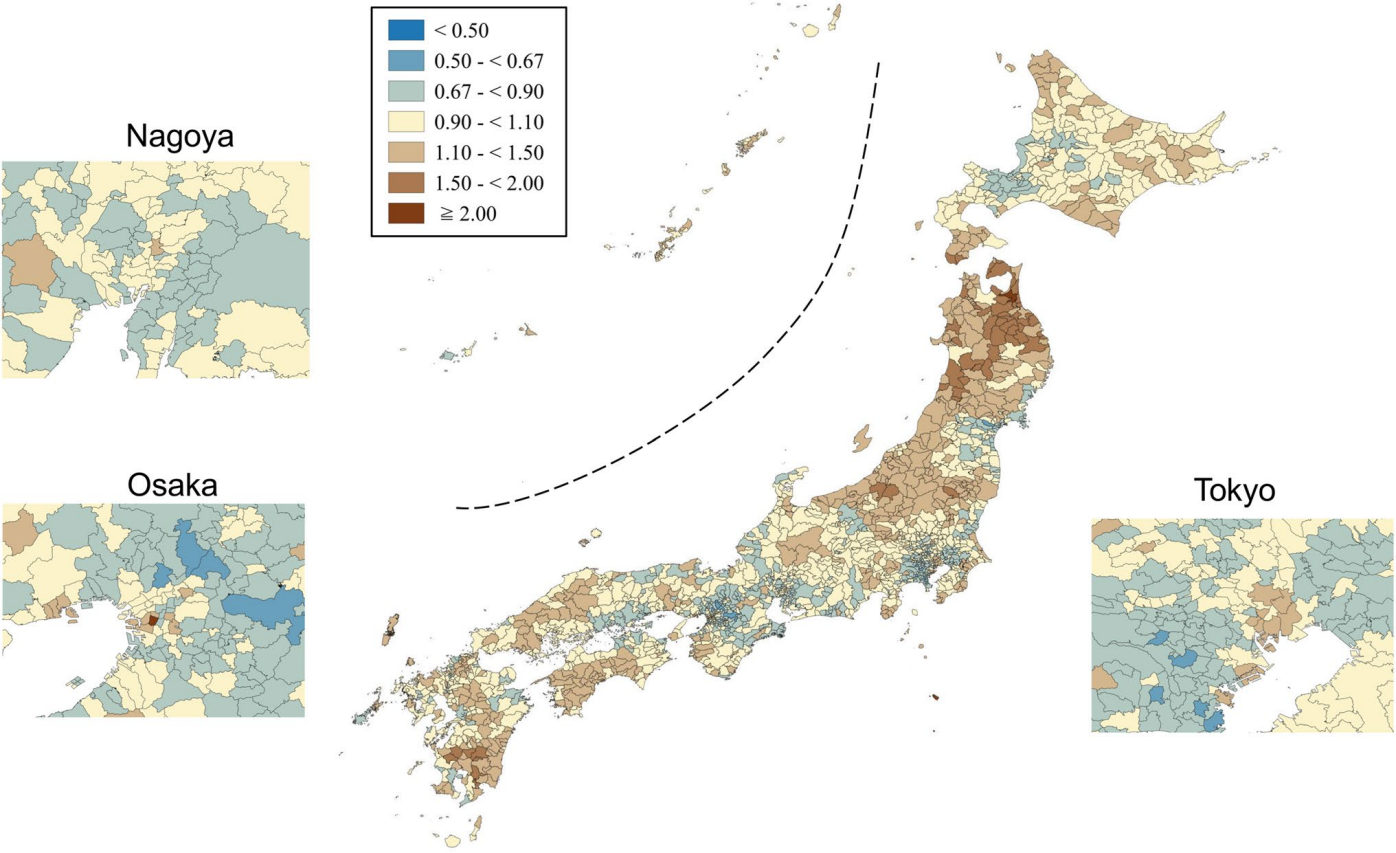
Maps of smoothed standardized mortality ratios (sSMRs) for suicide in males aged 60+ years across 1887 municipalities in Japan, 2009–2017

**Fig. 6:**
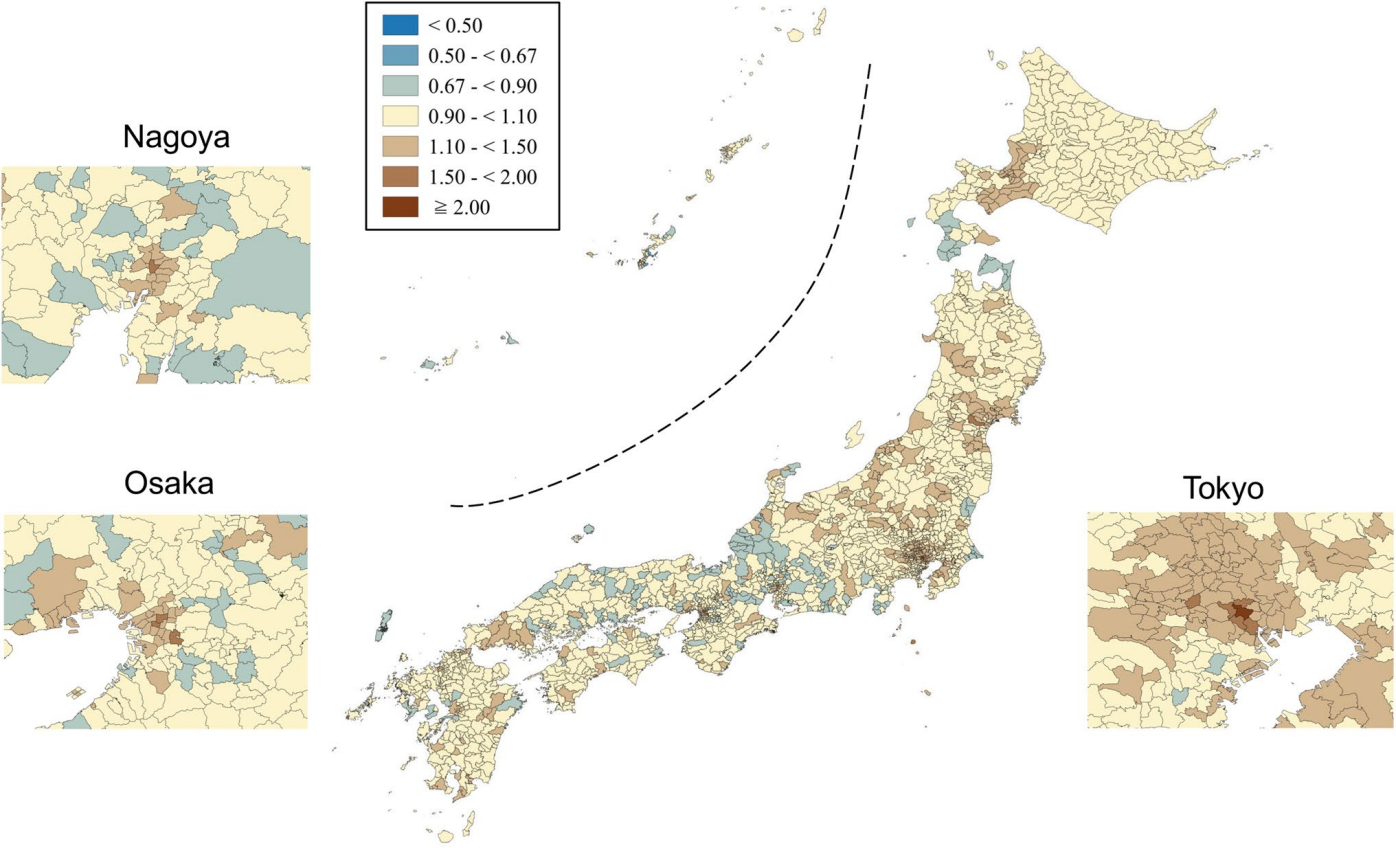
Maps of smoothed standardized mortality ratios (sSMRs) for suicide in females aged 0–39 years across 1887 municipalities in Japan, 2009–2017

**Fig. 7:**
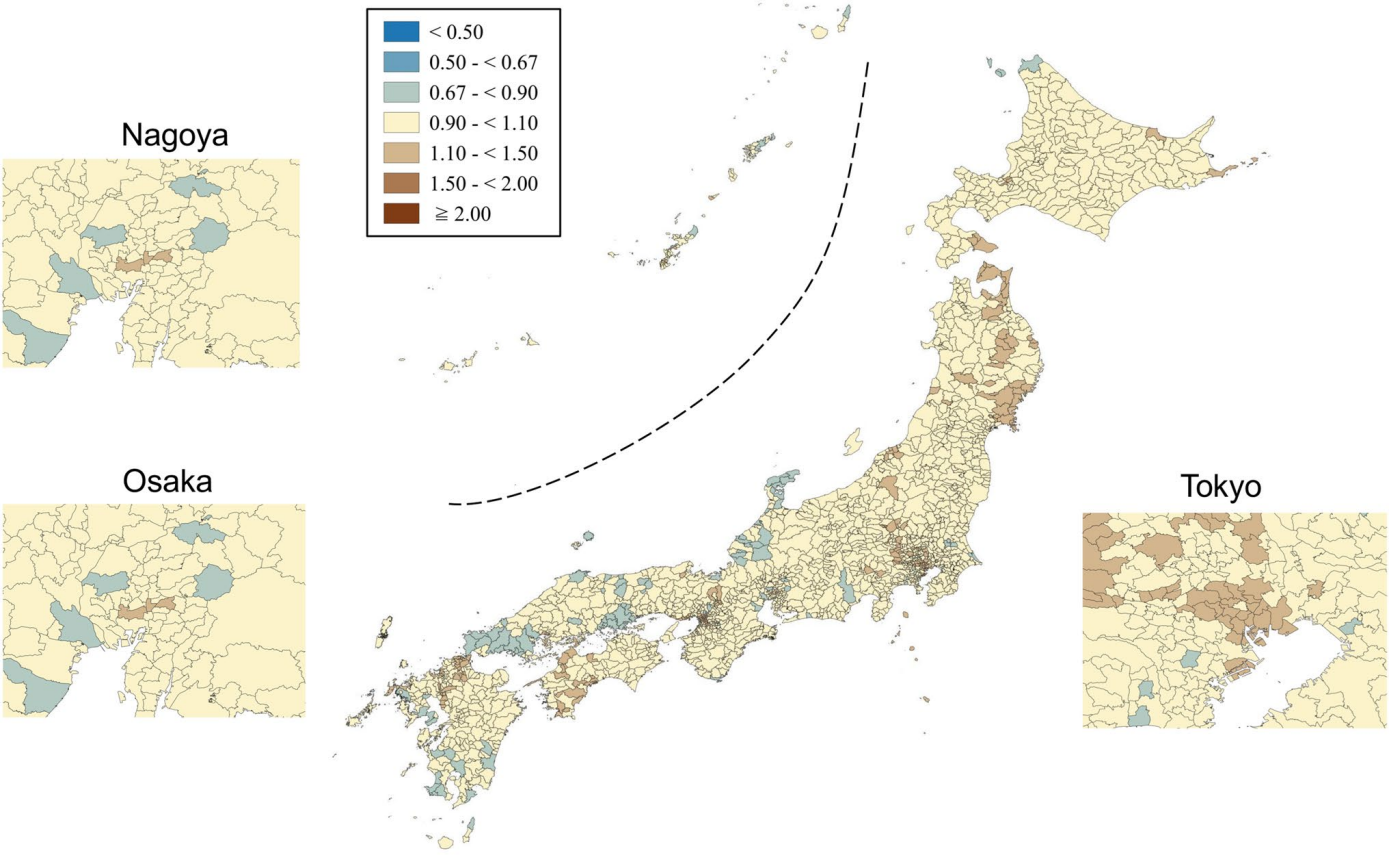
Maps of smoothed standardized mortality ratios (sSMRs) for suicide in females aged 40–59 years across 1887 municipalities in Japan, 2009–2017

**Fig. 8:**
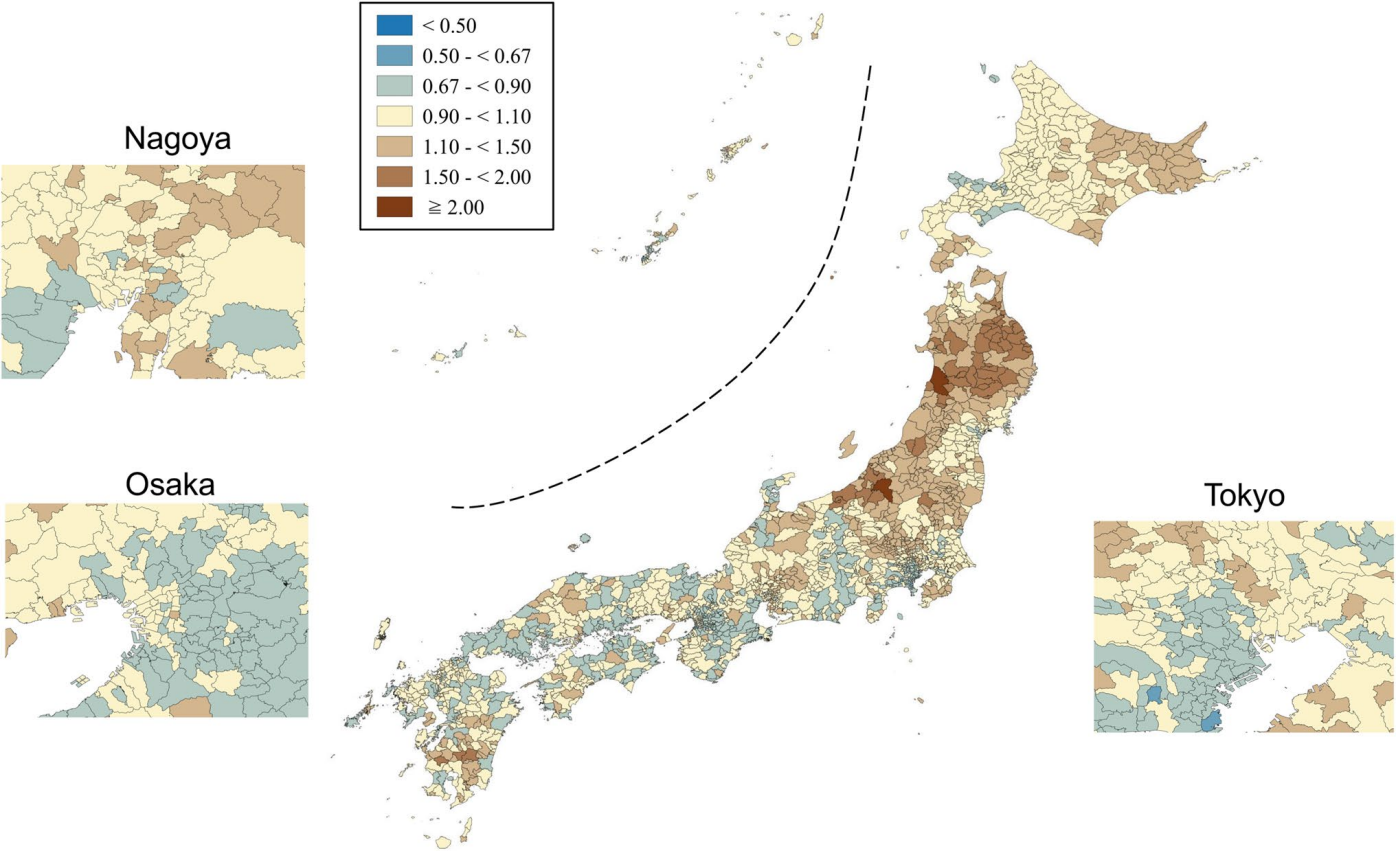
Maps of smoothed standardized mortality ratios (sSMRs) for suicide in females aged 60+ years across 1887 municipalities in Japan, 2009–2017

Appendix Figs. 9 and 10 show the maps of residual SMRs for males and females of all ages after considering rurality and socioeconomic characteristics, respectively. Although 90% ranges of the residual SMRs attenuated compared to those of the smoothed SMR, there was still a 1.3-fold difference in the 90% range for males of all ages (0.90–1.13) and a 1.4-fold difference for females of all ages (0.87–1.19). Compared to the smoothed maps, some of the concentrations of high-risk areas attenuated to a certain degree, while others remained high. This suggests that these high risks could not be explained fully by rurality/urbanity. Roughly decreasing trends were observed for both Japanese men and women, regardless of age and rurality level of the residence, during the research period (data were not shown).

**Fig. 9:**
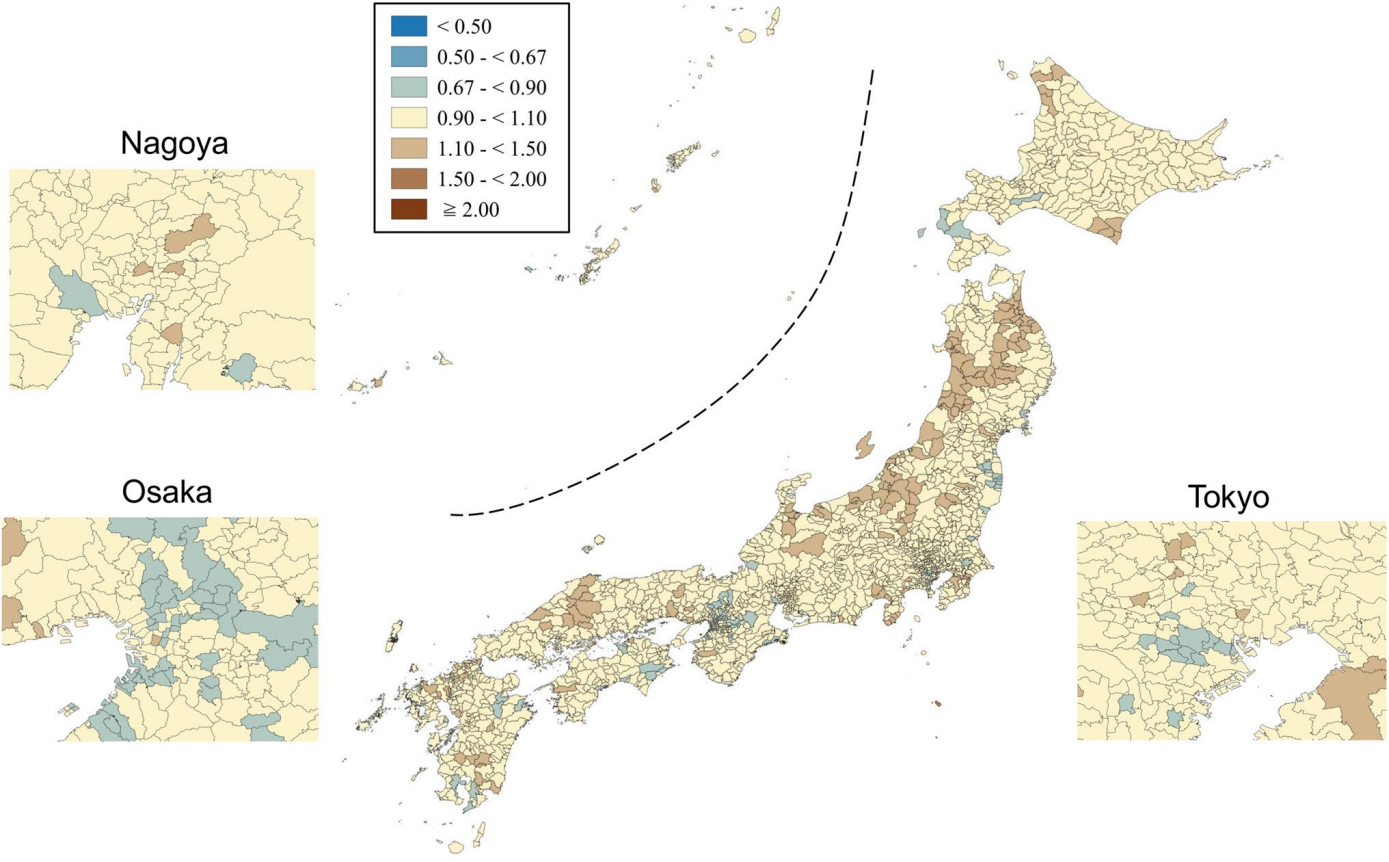
Maps of residual standardized mortality ratios (rSMRs) for suicide in males of all ages after controlling for rural–urban continuum and socioeconomic characteristics across 1887 municipalities in Japan, 2009–2017

**Fig. 10:**
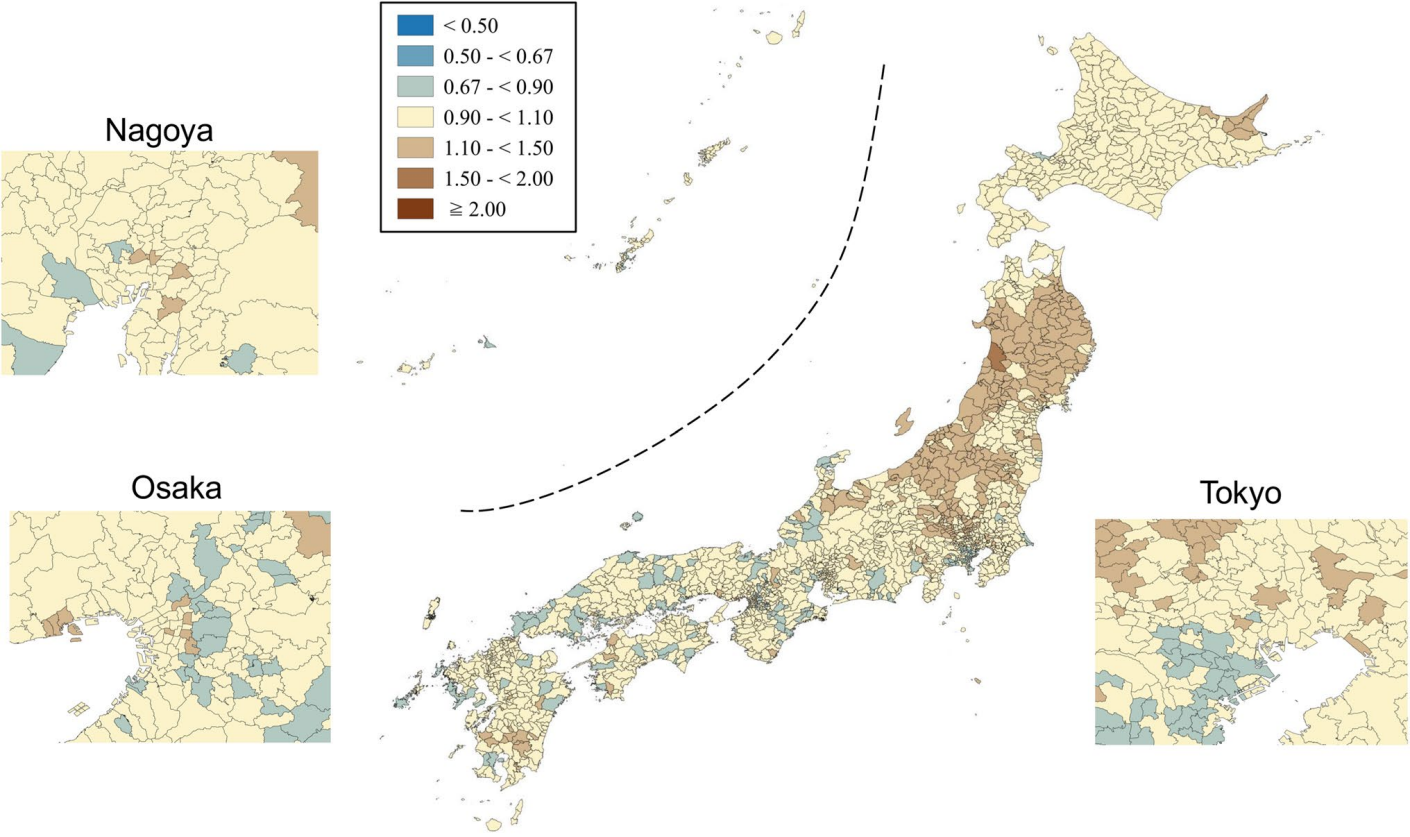
Maps of residual standardized mortality ratios (rSMRs) for suicide in females of all ages after controlling for rural–urban continuum and socioeconomic characteristics across 1887 municipalities in Japan, 2009–2017

## Discussion

### Main findings

This study examined the geographical distribution of suicide risk by gender and age group across 1887 municipalities in Japan, using 2009–2017 mortality data. Geographical distribution of suicide mortality in Japan differed substantially by gender and age with several regional clusters specific to some gender/age groups but not others. The most prominently clustered area of high suicide risk was the Tohoku region which is located in the northern part of the Honshu island. Clustering in this area was found among all gender/age groups except for females aged 0–39 and 40–59 years. Municipalities with low suicide risk tended to cluster in the three metropolitans for males, while those with high risk tended to cluster in those areas for females aged 0–39 years.

The results of the multivariate analyses showed that the geographical distribution of and rural–urban differences in suicide mortality in Japan differed substantially by gender and age. For males aged 0–39 and 40–59 years, rural residents tended to have a higher suicide risk compared to urban ones. For males aged 60+ years, a clear rural–urban gradient in suicide risk was not observed although residents with some categories of rurality had a significant higher risk. For females aged 0–39 years, no significant association between suicide risk and rurality was observed, while for females aged 40–59 years and 60 years or above, the association was U-shaped with a higher risk of suicide in rural and urban municipalities and a lower risk of suicide in one in the intermediate categories.

### Interpreting the findings

In this study, for males aged 0–39 and 40–59 years, rural residents had a higher suicide risk both before and after adjusting for socioeconomic variables. On the contrary, for males aged 60+ years, the difference in suicide risk between urban and rural municipalities almost disappeared after adjustment. These results indicate that the higher risk of suicide among rural residents can be largely explained by the socioeconomic variables considered here for males aged 60+ years, but not fully for those aged 0–39 and 40–59 years. A recent review article indicated that rural rates of suicidal behavior and death by suicide were often higher than those in urban areas [19], but some studies have reported that the association between suicide risk and rurality/urbanicity varies significantly by gender and age [15, 18]. Previous studies in Australia, Scotland, and USA reported that among rural residents, there was a higher risk of suicide among males, but not among females [10, 16, 20]. However, in the Netherland, Portugal, South Korea, and Taiwan, the risk was higher for both men and women [4, 5, 13, 21]. Concerning rural–urban differences by age, findings from the Netherland, South Korea, and Taiwan indicated that, regardless of age, suicide risk tended to be higher in rural areas than in urban areas [4, 13, 21]. On the other hand, research in Austria, Denmark, New Zealand, and the UK indicated that rural–urban differences in suicide risk varied by age [14, 15, 18, 22]. There are some possible explanations for increased suicide risk in rural areas [12, 19]. First, rural living can lead to social isolation, resulting in less intimate face-to-face contact with family and friends, which, in turn, increases suicide risk. Secondly, lack of access to mental health or emergency care, because of distance and a shortage of providers, can also be associated with an increase in suicide risk in rural areas [9, 36]. Similarly, in Japan, the presence of psychiatrists was significantly associated with reduced suicide risk in municipalities [11]. In addition, because of stigmatized attitudes toward visiting mental healthcare facilities, rural dwellers, particularly men, may be less willing to seek help for mental health problems than urban dwellers [19]. Thirdly, easier access to lethal means of suicide can lead to an increased suicide risk for rural dwellers. Firearms and pesticide ingestion are known to be suicide methods that occur at a higher rate in rural rather than urban areas [19]. However, recently in Japan, there have been very few suicides due to firearms and pesticides ingestion [37]. Therefore, we think that it is not possible that the availability of these two methods caused a higher suicide risk among Japanese men living in rural areas. Finally, socio-economic deprivation in rural areas may exert an adverse impact on the mental health of people living there. Recent finding of increasing rural suicide rates in many countries may reflect adverse socioeconomic trends in those areas, which have experienced slower economic development than urban areas, thereby leading to wider inequalities [19].

In this study, for females aged 0–39 years, the significant association between suicide risk and rurality disappeared after adjusting for socioeconomic variables, while for females aged 40–59 years and 60 years or above, the association was U-shaped after adjustment. These results indicate that rural–urban differences in suicide risk can be largely explained by the socioeconomic variables considered here for females aged 0–39 years in Japan, but not fully for those aged 40–59 years and 60+ years. Qin suggested that urban circumstances can have both adverse and beneficial effects on mental health of residents, and that women might be more vulnerable in urban competitive environments than their male counterparts [18]. In addition, rural-based characteristics, such as geographic and interpersonal isolation and lack of access to care, may also be a reason for the higher suicide risk in rural areas among women [19]. And thus, for middle-aged and older Japanese women, the adverse effects of both rural and urban areas on their mental health might contribute to the U-shaped association with a high suicide risk in both rural and urban areas.

The Japanese Government enacted the Basic Law on Suicide Prevention in 2006, and in turn began to take comprehensive national suicide prevention measures [38]. It was only after the enactment of this law that comprehensive measures against suicide, considering a wide range of social factors, were finally promoted at the national level. Under these suicide measures, the direction of the measures for each generation is indicated, taking into account the characteristics of suicide in each generation with respect to the three generations of young, middle-aged, and elderly people [39]. In addition, for each municipality to proceed with suicide measures that are suited to the local situation, the Japanese government has classified each municipality into three groups according to population size and has presented the basic policy package that is considered appropriate for each group [38]. These suicide prevention measures may have led to the decreasing suicide mortality rates across genders, ages, and levels of rurality/urbanity between 2009 and 2017, although whether and to what extent the suicide prevention measures were effective have not yet been fully investigated. Since we could not clarify how differences in suicide rates between rural and urban areas in Japan changed during the research period, further studies are needed to examine trends in rural–urban differences in suicide mortality rates and the association, if any, with suicide prevention measures in Japan.

### Implications for public health policies and future researches

Our findings have important implications for the national planning of suicide prevention activities in Japan and the other countries. Our results showed that there were rural–urban differences in suicide mortality in Japan in the period 2009–2017, but that the differences varied substantially according to gender and age. These results indicate that, to appropriately allocate resources for suicide prevention in municipalities, demographic factors need to be taken into consideration. One previous study in Japan reported that comprehensive community suicide countermeasures were effective in lowering suicide rates in rural areas but not in urban areas, and that the countermeasures were more effective for males than females and for the elderly than the young [40]. This may indicate that lowering the suicide rate among young Japanese women living in urban areas is a more difficult problem to address compared with other gender/age groups living in rural or urban areas, although the suicide risk for young Japanese women was higher in urban than in rural areas. And therefore, we think that further examination into the determinants of suicide and implementation of suicide countermeasures tailored to actual conditions among young Japanese women in urban areas should be high priority issues.

Findings from Taiwan showed that there were marked differences in geographic distributions of suicide according to suicide methods used, and that such differences are thought to be mainly due to differences in accessibility to lethal methods of suicide [4]. This indicates that the choice of suicide method could vary to some degree between rural and urban populations and be partially responsible for rural–urban differences in suicide mortality rates. Since restricting access to lethal means of suicide is one of the few prevention measures supported by the available evidence [41], an examination of spatial patterns of method-specific suicide rates may contribute to reducing the geographical disparities in suicide rates to some extent.

Finally, geographic variations in suicide could be associated with regional socio-economic characteristics, such as socio-economic deprivation, social fragmentation and access to mental health services [6, 30], and thus further research into the associations between regional suicide risk and their characteristics may have important implications for tackling higher suicide rates in disadvantaged areas.

## Limitations

Our study had several methodological issues which must be acknowledged. First, we used municipalities as the unit of analysis. Because the municipalities in Japan vary greatly in both geographical and population sizes, the suicide pattern within one particularly large municipality may not be homogenous. In addition, because inland municipalities tend to have more neighboring municipalities than coastal ones, it is possible that some inland municipalities are over-smoothed [10]. Also, some suicide cases had to be omitted, since they could not be assigned to municipalities due to lack of residential data. However, since only 1.1% of suicides were omitted in this study, so we believe that this exclusion would not significantly affect spatial patterns of suicide risk presented in this article.

Secondly, in this study, we used population density as an indicator of level of rurality/urbanity. Rurality/urbanicity has been frequently assessed by population density in epidemiological studies, since the data for calculating population density are easily accessible, annually updated, and available in most countries, which facilitates inter-country comparability between studies [4, 12, 13, 22]. However, population density is only a place-based representation and does not consider interaction with other areas [12]. And thus, further studies are needed to go beyond a single representation of rurality/urbanicity when exploring suicide inequalities spatially in Japan.

Thirdly, during the 9-year study period, national suicide rates in Japan decreased by approximately 30% for both males and females of all ages; different municipalities might have experienced different secular trends in suicide, and thus the geographic pattern may have changed over time. However, the small number of suicides at the municipality level required aggregating data over several years to ensure sufficient incidence in each area, particularly when conducting gender- and age-specific analyses.

Fourthly, we used suicide statistics instead of cause-of-death statistics in this study. Suicide statistics are compiled by the police agency based on data on unnatural deaths, while cause-of-death statistics are compiled by the Ministry of Health, Labour, and Welfare based on data from death certificates. There is a slight difference in the number of deaths between these two statistics due to how unidentified causes of deaths are handled and those of non-Japanese nationalities.

Fifthly, since this is an ecological study, the associations identified cannot be directly inferred at the individual level.

Finally, congruent with most previous studies [3–5, 9, 10], we assumed that people are only exposed to their actual place of residence. As suicide risk develops over a lifetime, future studies should be longitudinal and include people’s residential history over their life course.

## Data Availability

All data used in this manuscript are publicly available. Suicide data are available from the website of suicide statistics (https://www.mhlw.go.jp/stf/seisakunitsuite/bunya/0000140901.html). Data about population estimate are available from the website of Statistics Japan (https://www.e-stat.go.jp/stat-search/files?page=1&toukei=00200241&tstat=000001039591). Data about Census in 2010 are available from the website of Statistics Japan (https://www.e-stat.go.jp/stat-search/files?page=1&toukei=00200521&tstat=000001039448). Code availability: Statistical analyses were performed using the R-INLA library (18.07.12) in R-3.5.3 and Stata statistical software, version 15.1, for Macintosh (StataCorp, College Station, TX, USA). All maps were produced using QGIS Version 2.18.15 for Macintosh.

## Appendix

See Figs. 3, 4, 5, 6, 7, 8, 9, 10 and Table 4.
